# Supplementary material for: Hyphal compartmentalization and sporulation in Streptomyces require the conserved cell division protein SepX
Source: Nat Commun. 2022 Jan 10;13:71. doi: 10.1038/s41467-021-27638-1 (PMC8748795; doi:10.1038/s41467-021-27638-1)
Supplement: Supplementary file 1 — Supplementary Information [file 41467_2021_27638_MOESM1_ESM.pdf]

Supplementary Information

Hyphal compartmentalization and sporulation in *Streptomyces* require the conserved cell division protein SepX

Authors

Matthew J. Bush[1], Kelley A. Gallagher[1,3], Govind Chandra[1], Kim C. Findlay[2] and Susan Schlimpert[1]*

Affiliations

[1] Department of Molecular Microbiology, John Innes Centre, Norwich Research Park, Norwich NR4-7HU UK

[2] Department of Cell and Developmental Biology, John Innes Centre, Norwich Research Park, Norwich, NR4-7HU, UK

[3] Département de Microbiologie, Infectiologie et Immunologie, Université de Montréal, Pavillon Roger-Gaudry, 2900, boulevard Édouard-Montpetit, C.P. 6128, Succursale Centre-ville, Montréal, QC H3C 3J7, Canada

* Correspondence: susan.schlimpert@jic.ac.uk

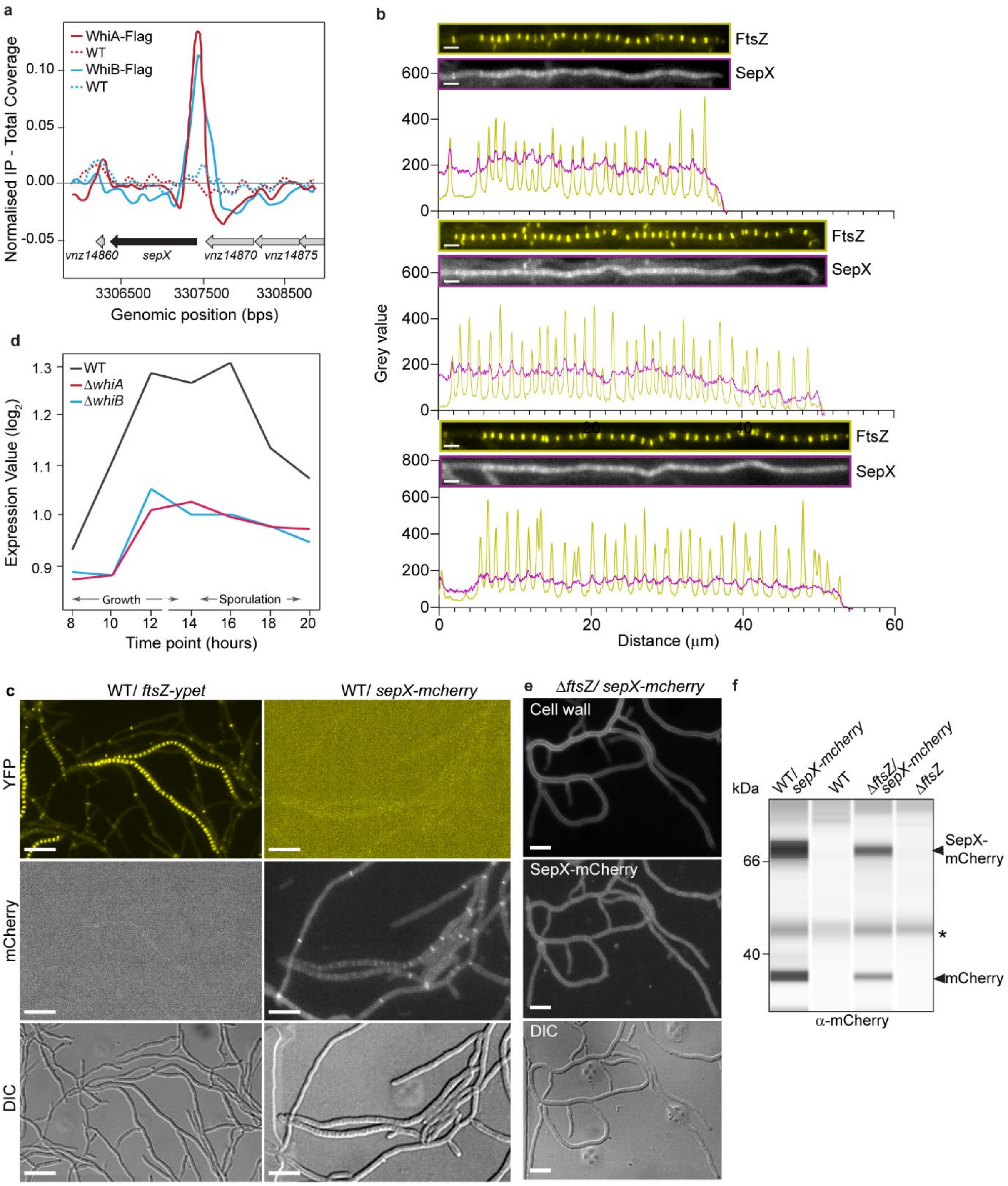

Supplementary Figure 1: SepX is developmentally regulated and requires FtsZ for localization. (a) *sepX* (*vnz_14865*) is a WhiAB-target as identified by ChIP-seq. Enrichment (normalized IP – total coverage) in strains expressing either WhiA-FLAG (solid red line) or WhiB-FLAG (solid blue line) but not in the untagged wild type controls (dashed red and dashed blue lines) was observed upstream of the *sepX* gene *in vivo*[14,15]. **(b)** Additional fluorescence intensity profiles of FtsZ-YPet and SepX-mCherry in sporulating hyphae shown in Supplementary Movie 1. Scale bars: 2 μm. Data shown are representatives of four independent hyphae. Source data are provided as a Source Data file. **(c)** Control images showing the absence of spectral bleedthrough and the specificity of the FtsZ-YPet and SepX-mCherry fluorescence signal using the same illumination settings (strains LUV015, MB1124). Scale bars: 5 μm. Data shown are representatives of at least 10 images. **(d)** WhiA and WhiB co-activate

the transcription of *sepX.* Data represent transcriptomic data during submerged sporulation in wild-type *S. venezuelae* (black line); the congenic *whiA* mutant (red line); and the congenic *whiB* mutant (blue line)[14,15,19]. The x-axis indicates the age of the culture in hours, and the y-axis indicates the per-gene normalized transcript abundance (Expression Value log_2). **(e)** Microscopic analysis of SepX-mCherry distribution in the Δ*ftsZ* mutant background (MB1082). Hyphal cell wall was visualized using 0.25 mM HADA. DIC, differential interference contrast. Scale bar: 5 μm. Data shown are representatives of at least 10 images. **(f)** Virtual Western blot showing SepX-mCherry abundance in the wild type (MB1124) and the Δ*ftsZ* mutant (MB1082) compared to the corresponding untagged strains. Automated western blot analysis was performed in biological duplicate. Equal amounts of protein lysate were loaded and SepX-mCherry was detected using α-mCherry antibody. Asterisk denotes an unspecific signal detected in all samples.

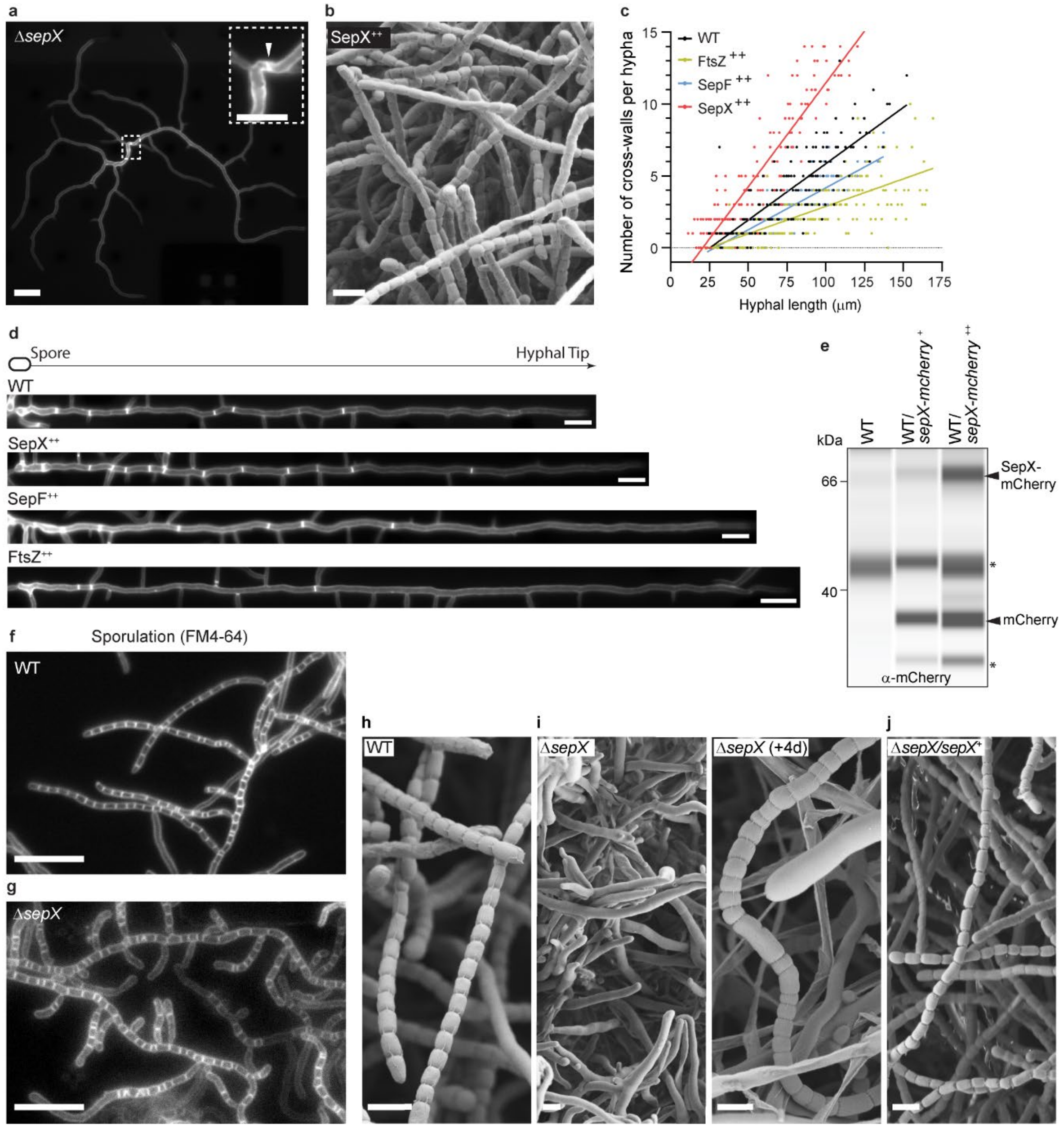

Supplementary Figure 2: SepX is a determinant of cross-wall formation and is required for regular sporulation. (a) Representative image of HADA-stained hyphae emerging from a Δ*sepX* spore (small dashed box) Scale bar: 10 μm. Occasionally, a division septum can be detected close to the mother spore (magnified region in inset). Scale bar: 5 μm. Experiments were performed in triplicate. **(b)** Cryo-SEM image of spore chains produced by wild-type *S. venezuelae* constitutively expressing *sepX* (MB168, SepX^{++}). Scale bar: 2 μm. (Representative of least 7 images) **(c)** Quantification of cross-wall frequency in strains constitutively expressing *sepX* (SepX^{++}, MB168), *ftsZ* (FtsZ^{++}, MB127) or *sepF* (SepF^{++}, SS414) compared to the wild type (WT). Strains were allowed to germinate and grow in the presence of 0.25 mM HADA to visualize cross-walls. Quantification is based on biological triplicate experiments per strain. Hyphae emerging from at least 20 spores per replicate were analyzed. Solid line represents simple linear regression. **(d)** Representative images of straightened hyphae showing the distribution of cross-walls in the same strains analyzed in (c). Scale bars: 5 μm. Experiments were performed in triplicates. **(e)** Virtual Western blot showing SepX-mCherry levels when produced from

the native promoter (+) or a constitutive promoter (++). Equal amounts of proteins were loaded and SepX-mCherry was detected using an α-mCherry antibody. Asterisks denote unspecific signals and cleavage products. Shown are representative data from duplicate experiments. **(f)** and **(g)** Fluorescent micrographs of sporulating wild-type (WT) and Δ*sepX* (SV55) hyphae stained with FM4-64 to visualize sporulation septa. Scale bar: 10 μm. Shown are representative hyphae of at least 5 images. **(h-j)** Cryo-SEM images showing sporulation septation in hyphae of the wild type (WT, h), the Δ*sepX* mutant (SV55, i) and the complemented mutant (MB181, j). Strains were grown on solid MYM and colonies imaged after three days (representative of at least seven images). The Δ*sepX* mutant does not sporulate after three days but eventually forms irregular spore chains after additional four days of incubation. Scale bars: 2 μm.

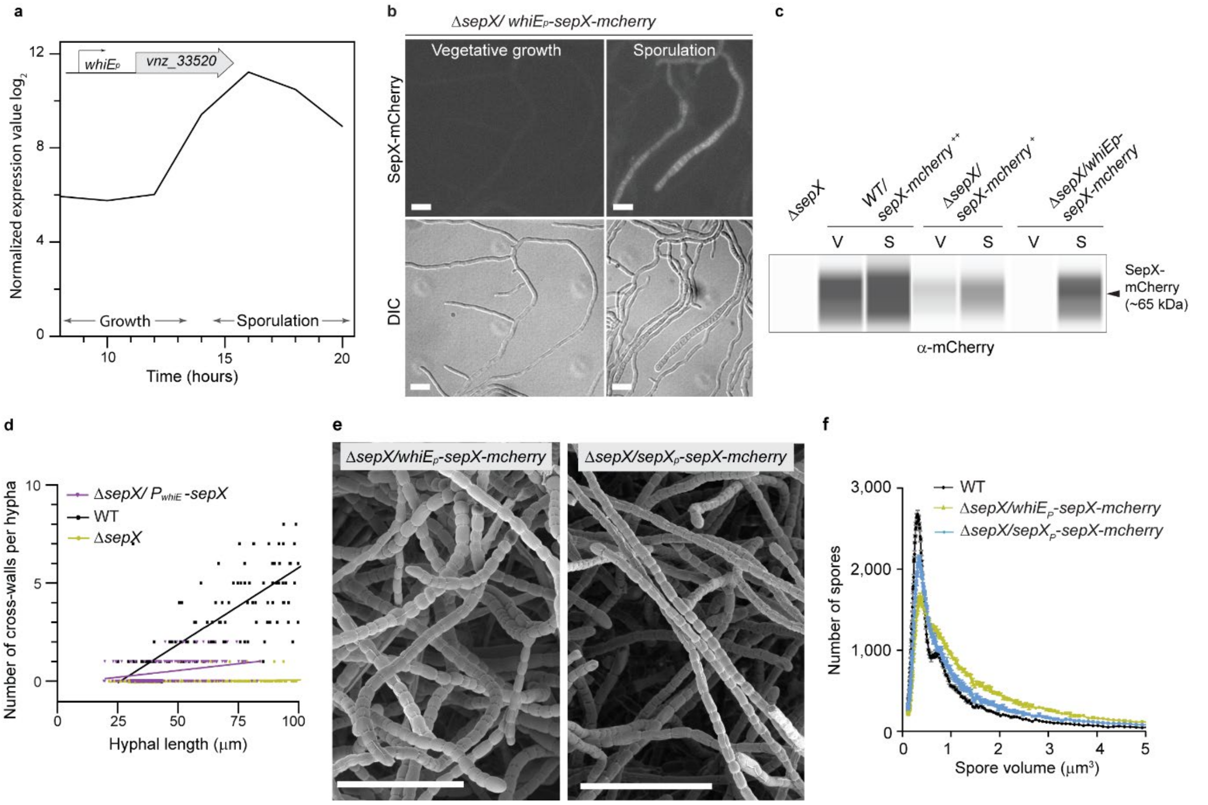

Supplementary Figure 3: Sporulation-specific expression of *sepX* results in a largely absence of cross-walls but normal sporulation septation. (a) Expression profile showing the sporulation-specific activity of *whiE$_P$*-driven gene expression over the *S. venezuelae* life cycle based on microarray data for *vnz_33520*. Transcriptomic source data was obtained from Al-Bassam et al. (2014)[1]. **(b)** Microscopic analysis of SepX-mCherry localization following expression from the *whiE* promoter in the Δ*sepX* mutant background (MB1120). Septal localization of SepX-mCherry is only observed during sporulation. Scale bars: 5 μm. Shown are representative of at least five images. Source data are provided as a Source Data file. **(c)** Virtual Western blot showing the abundance of SepX-mCherry during vegetative growth (V) and sporulation (S). SepX-mCherry levels were determined in the wild type expressing *sepX* from the *ermE** constitutive promoter (++, MB1124) and in the Δ*sepX* mutant (SV55), either from the native promoter (+, MB171) or the *whiE*-promoter (MB1120). SepX-mCherry was first immunoprecipitated from whole cell lysates. Equal total protein from each strain was used as input for the immunoprecipitation and equal volume subsequently loaded for Western analysis following enrichment. Protein levels were analyzed using an α-mCherry antibody. Experiments were performed in biological duplicate. **(d)** Quantification of cross-wall frequency in the Δ*sepX* mutant expressing *sepX* from the *whiE*-promoter (MB1120). Experiments were performed as described above. Hyphae of at least 20 spores per biological replicate (n=3) were analyzed. For comparison, cross-wall distribution of the wild type (WT) and the Δ*sepX* mutant (SV55) from Figure 2c are shown again. Solid line represents simple linear regression. **(e)** Cryo-SEM images of spore chains in the Δ*sepX* mutant complemented with *sepX-mcherry* expressed from the *whiE* promoter (left, MB1120) or the native promoter (right, MB171). Scale bar: 10 μm. Shown are representative of at least seven images per strain. **(f)** Spore volume frequency distributions (normalized to total count, n=100,000) of the wild type (WT) and the Δ*sepX* mutant complemented with *sepX-mCherry* expressed from the *whiE* promoter (MB1120) or its native promoter (MB171). Shown is the mean ± s.e.m. of three biological replicates per strain. Source data are provided as a Source Data file.

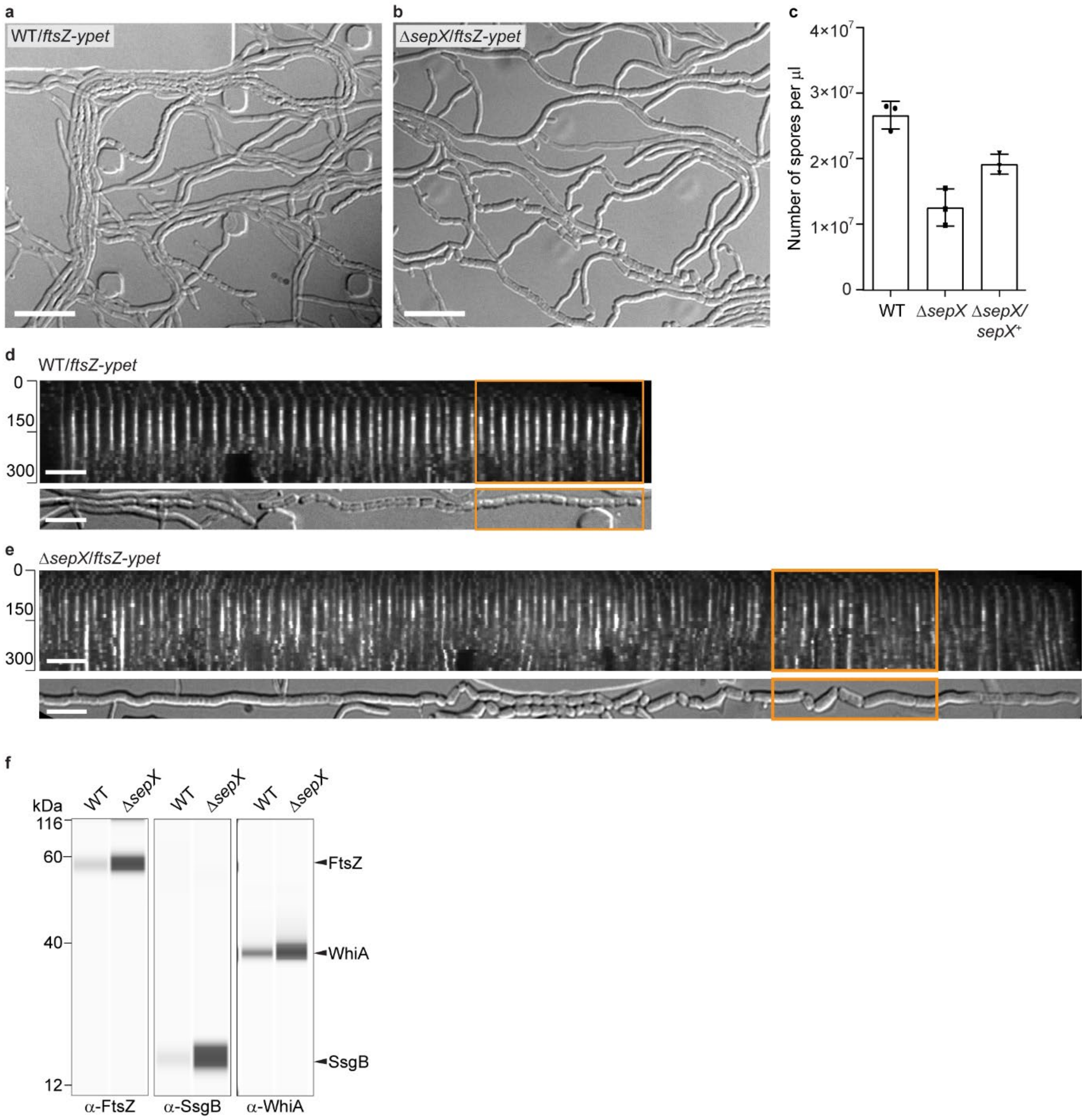

Supplementary Figure 4: SepX is required for wild type-like sporulation. (a) and **(b)** are differential interference contrast (DIC) images corresponding to the fluorescent micrographs shown in Figure 4a and b. Note that the images show the outcome of the sporulation septation process in (a) the wild type (SS12) and (b) the Δ*sepX* mutant (MB180). Scale bars: 10 µm. **(c)** Number of spores produced by the wild type (WT), the Δ*sepX* mutant (SV55) and the complemented mutant (MB181). Shown is the mean ± s.e.m. Source data are provided as a Source Data file. **(d)** and **(e)** Representative kymographs of FtsZ-YPet fluorescence and the corresponding DIC images obtained from the fluorescence time-lapse image series (n=5 per strain) of wild-type (SS12) and *sepX*-deficient hyphae (MB180). The orange boxes indicate the subsections shown in Figure 4c and 4d. Scale bars: 5 µm. **(f)** Virtual Western blot showing the abundance of FtsZ, SsgB and WhiA in the wild type (SS12) and the Δ*sepX* mutant (MB180) during sporulation. Samples were taken from sporulating cultures and proteins were detected using α-FtsZ, α-SsgB and α-WhiA polyclonal antibodies respectively. The data shown here are representative for experiments performed in duplicate or in triplicate (c).

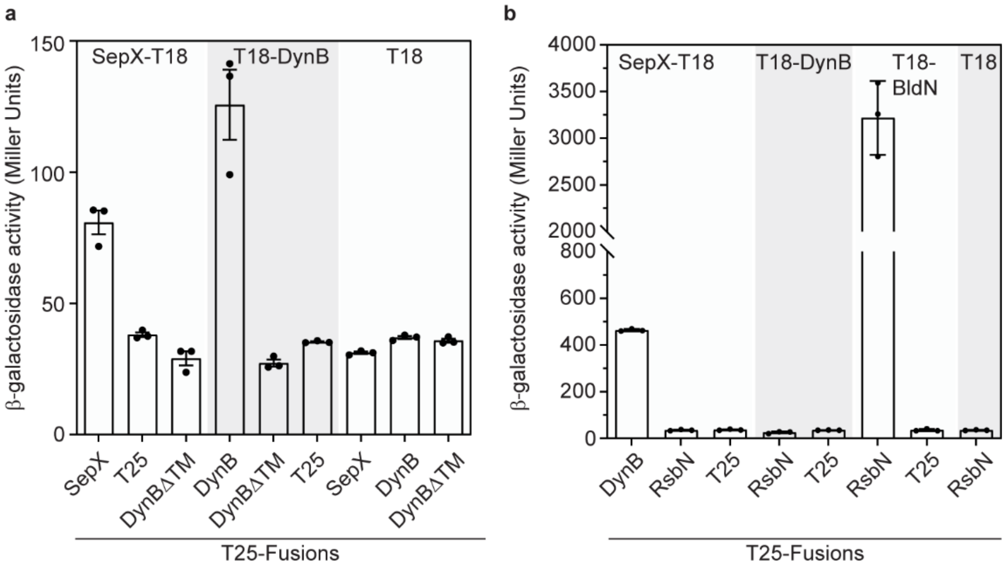

Supplementary Figure 5: SepX specifically interacts with DynB via the DynB transmembrane segment. Additional bacterial two-hybrid interaction studies. β-galactosidase activity was obtained from three biological replicates showing in **(a)** the self-interaction of SepX and loss of interaction with DynB carrying a deletion of the two transmembrane segments (DynBΔTM) and in **(b)** that SepX specifically interacts with DynB but not with other membrane proteins like BldN. Data points in (a) and (b) present mean values +/- s.e.m. Each interaction was assayed in triplicate.

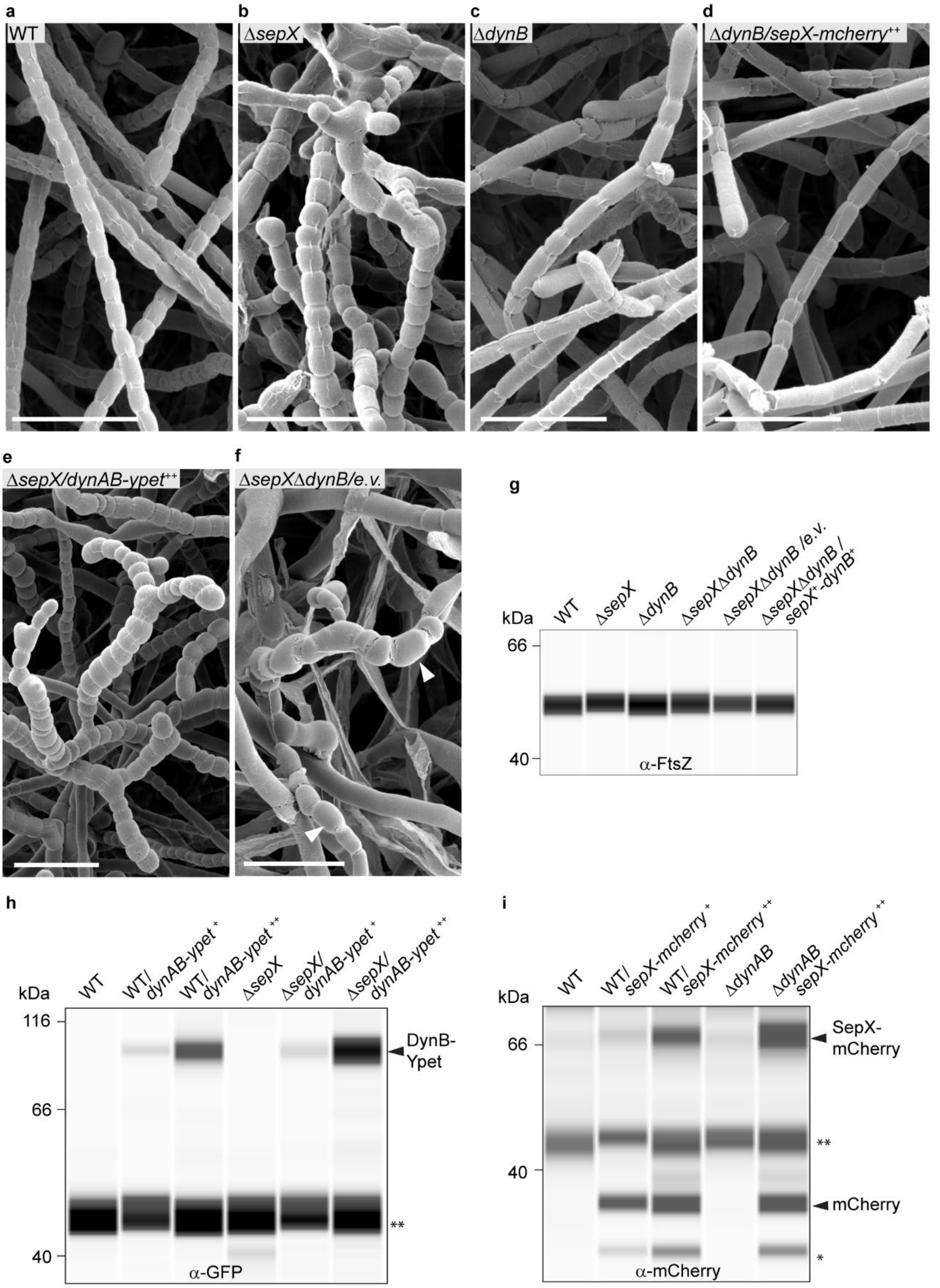

Supplementary Figure 6: SepX and DynB function independently during sporulation. Cryo-SEM images showing the terminal sporulation phenotype of **(a)** the wild type (WT), **(b)** the Δ*sepX* mutant (SV55), **(c)** the Δ*dynB* mutant (SS2), **(d)** the Δ*dynB* mutant constitutively expressing *sepX-mcherry* (++, MB1092), **(e)** the Δ*sepX* mutant constitutively *dynAB-ypet* (++, MB1175) and **(f)** the Δ*sepX*Δ*dynB* double mutant carrying an empty vector (e.v., MB1099). White arrow heads in (f) point at the rare and

aberrant spore chains. Scale bars: 5 μm. **(g)-(i)** Virtual Western blots showing the abundance of **(g)** FtsZ in Δ*sepX* (SV55), Δ*dynB* (SS2), Δ*sepX*Δ*dynB* (SV57) and the complemented mutant strain expressing both *sepX* and *dynB* (MB1103) or carrying the empty plasmid (MB1099); **(h)** DynB-YPet produced either from the native (+) or a constitutive (++) promoter in the wild type (WT), SS92, MB1180, SV55, SS155 and MB1175 strains; **(i)** SepX-mCherry produced either from the native (+) or a constitutive (++) promoter in the WT, MB170, MB1124, LUV001 and MB1092 strains. Protein levels were verified using α-FtsZ, α-GFP and α-mCherry antibodies respectively. Asterisks denote unspecific cross-reactions of the antibody (**) or degradation products (*). Data shown in (a)-(f) are representative of at least seven images per strain. Western blots shown in (g)-(i) were performed in duplicate.

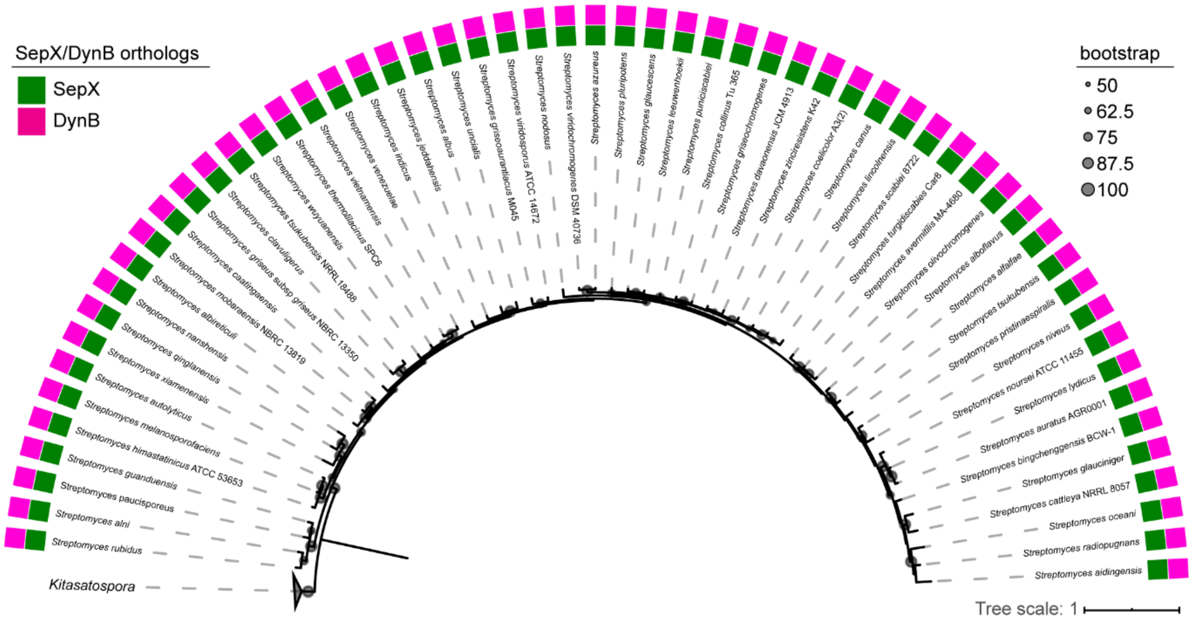

Supplementary Figure 7: SepX and DynB are highly co-conserved in *Streptomyces*. Distribution of SepX and DynB homologs in the genus *Streptomyces*. Maximum likelihood phylogeny of 58 representative *Streptomyces* genomes, based on concatenated alignment of 37 housekeeping genes. Tree is derived from the analysis presented in Supplementary Figure 9 with the *Streptomyces* clade un-collapsed. Bootstrap values >50% are indicated by grey circles at each node. Green and magenta boxes are used to represent the presence of SepX/DynB orthologs within each genome. Units of the tree are substitutions per site.

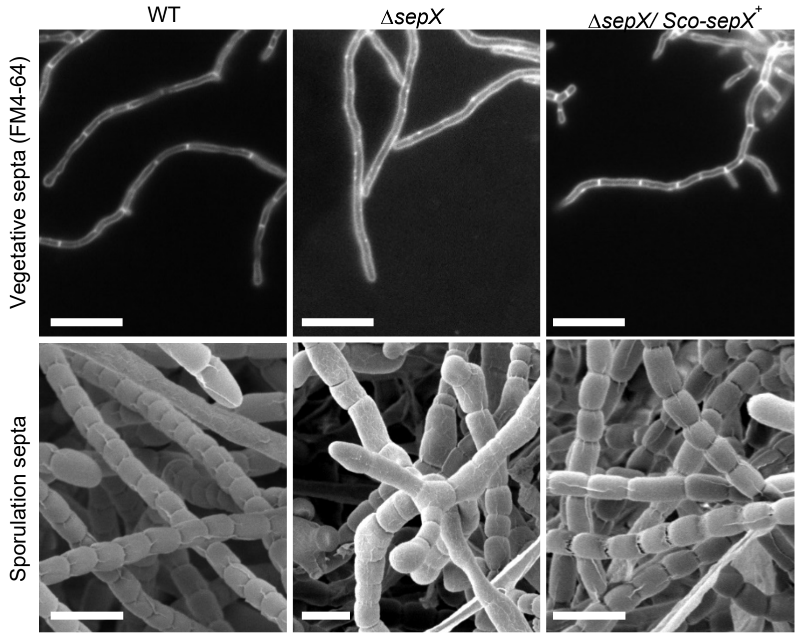

Supplementary Figure 8. Cross-complementation experiment. Expression of *sepX* from *S. coelicolor in trans* in the *S. venezuelae* Δ*sepX* mutant (MB1104) can restore vegetative septa formation and wild type-like sporulation in the Δ*sepX* mutant (SV55). Vegetative septa were visualized using the fluorescent membrane dye FM4-64 (scale bars 10 μm). Spore chains were images by cryo-SEM (scale bars: 2 μm). Representative of at least five images are shown.

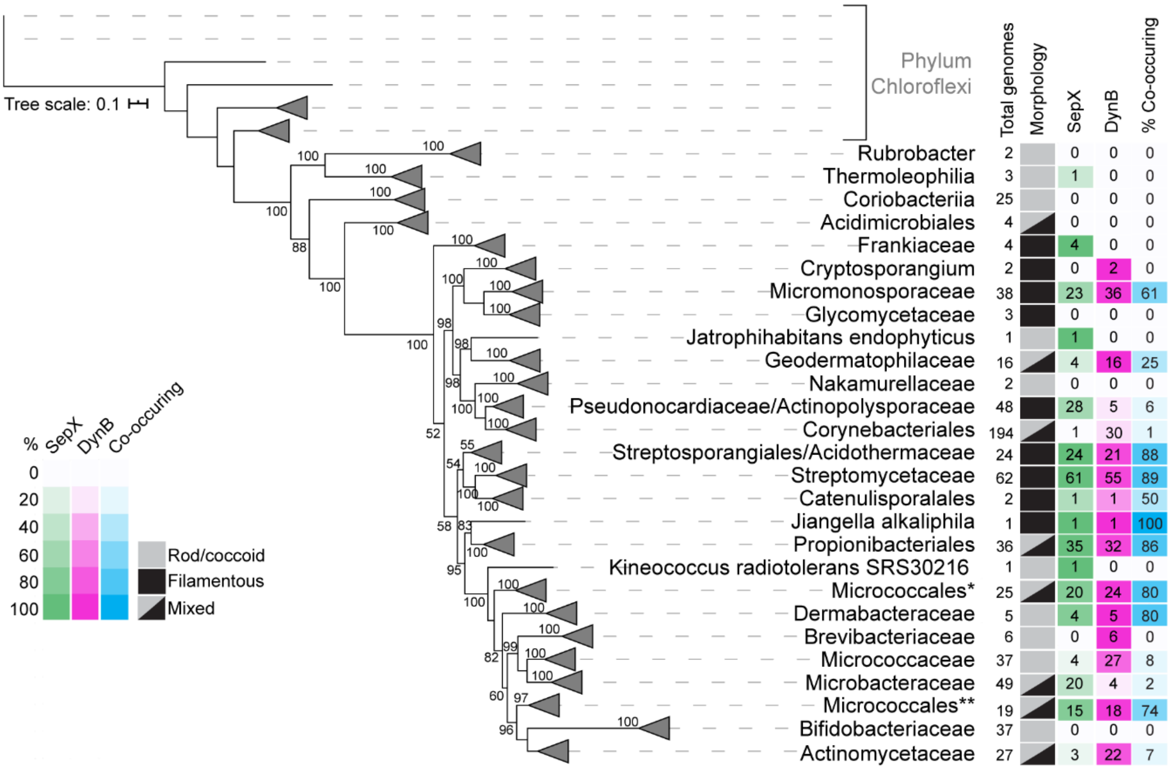

Clade	Total genomes	SepX	DynB	% Co-occuring
Rubrobacter	2	0	0	0
Thermoleophilia	3	1	0	0
Coriobacteriia	25	0	0	0
Acidimicrobiales	4	0	0	0
Frankiaceae	4	4	0	0
Cryptosporangium	2	0	2	0
Micromonosporaceae	38	23	36	61
Glycomycetaceae	3	0	0	0
Jatrophihabitans endophyticus	1	1	0	0
Geodermatophilaceae	16	4	16	25
Nakamurellaceae	2	0	0	0
Pseudonocardiaceae/Actinopolysporaceae	48	28	5	6
Corynebacteriales	194	1	30	1
Streptosporangiales/Acidothermaceae	24	24	21	88
Streptomycetaceae	62	61	55	89
Catenulisporalales	2	1	1	50
Jiangella alkaliphila	1	1	1	100
Propionibacteriales	36	35	32	86
Kineococcus radiotolerans SRS30216	1	1	0	0
Micrococcales*	25	20	24	80
Dermabacteraceae	5	4	5	80
Brevibacteriaceae	6	0	6	0
Micrococcaceae	37	4	27	8
Microbacteraceae	49	20	4	2
Micrococcales**	19	15	18	74
Bifidobacteriaceae	37	0	0	0
Actinomycetaceae	27	3	22	7

Supplementary Figure 9: Distribution of SepX and DynB homologs in the Phylum Actinobacteria. Data is presented as a maximum likelihood phylogenetic tree based on 673 representative actinobacterial genomes. Ten genomes from Phylum Chloroflexi are included as outgroups. Bootstrap values ≥50% are labeled at their respective nodes (values from 50-74% are indicated with †, 75-89% with ‡, and 90-100 with •). Triangles are used to represent where clades have been collapsed. For each clade, the total number of genomes, the number of genomes possessing a SepX and/or DynB ortholog, and the percentage of genomes where SepX/DynB co-occur are provided. Heatmaps are used to represent the percentage of total genomes within each clade possessing a SepX/DynB ortholog. Morphology designations for each clade are based on literature reports. *Includes Micrococcales families Dermatophilaceae, Intrasporangiaceae, Dermacoccaceae. **Includes Micrococcales families Promicromonosporaceae, Cellulomonadaceae, Sanguibacteraceae, Jonesiaceae, Ruaniaceae, and Beutenbergiaceae. Units of the tree are substitutions per site.

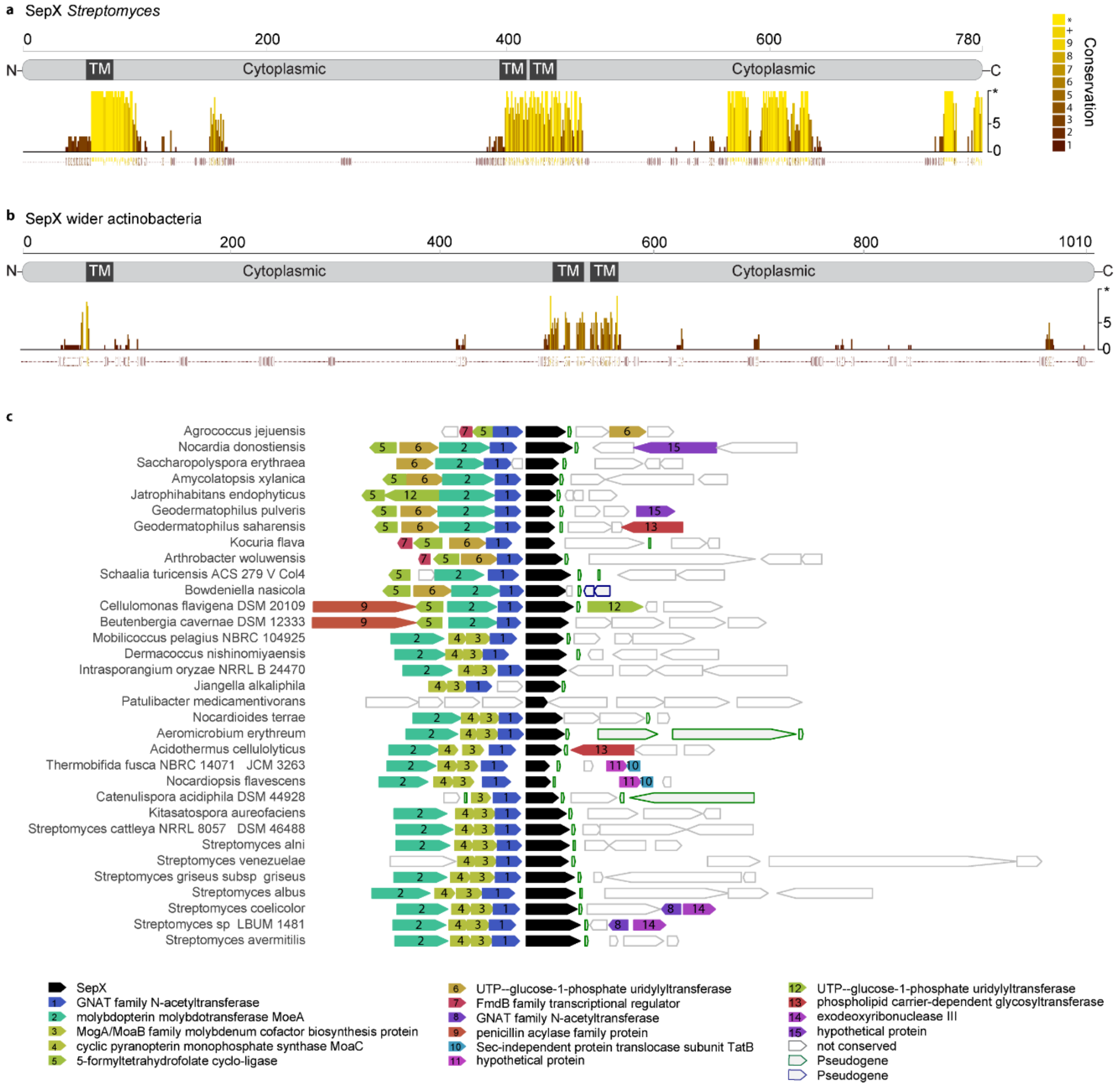

Supplementary Figure 10: Sequence and gene neighborhood conservation of SepX homologs. (a-b) Sequence conservation of SepX homologs from (a) *Streptomyces* (n=58) and (b) the wider actinobacteria (n=251). Conservation was calculated based on the Analysis of Multiply Aligned Sequences (AMAS) method implemented in Jalview[39]. **(c)** Gene neighborhood of *sepX* (shown in black) from 32 well-distributed representatives actinobacterial genomes that were selected based on phylogeny.

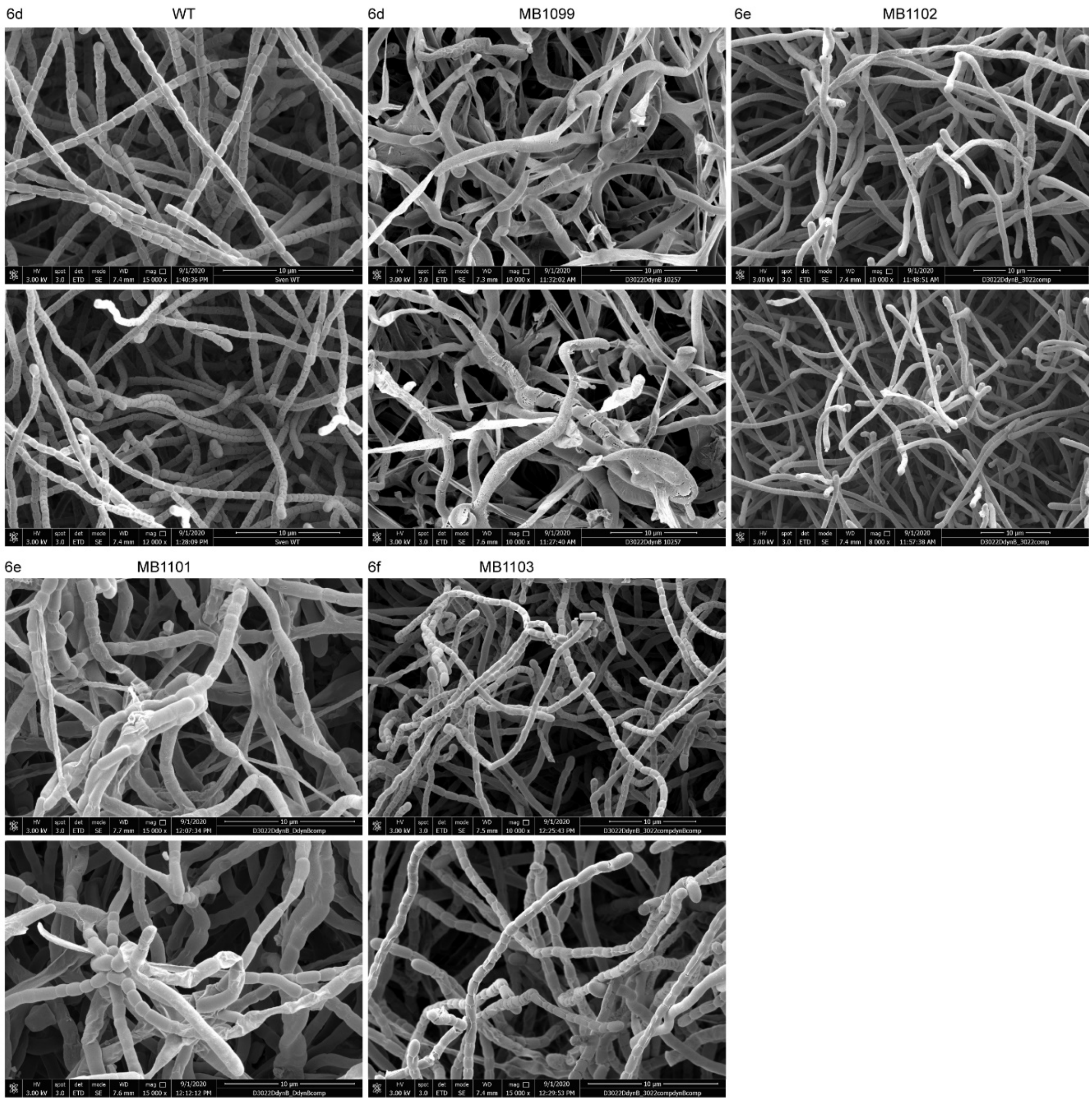

Supplementary Figure 11: Full uncropped SEM images shown in Figure 6. Numbers correspond to figure panels shown in Figure 6, additional representative SEMs for each strain are shown below.

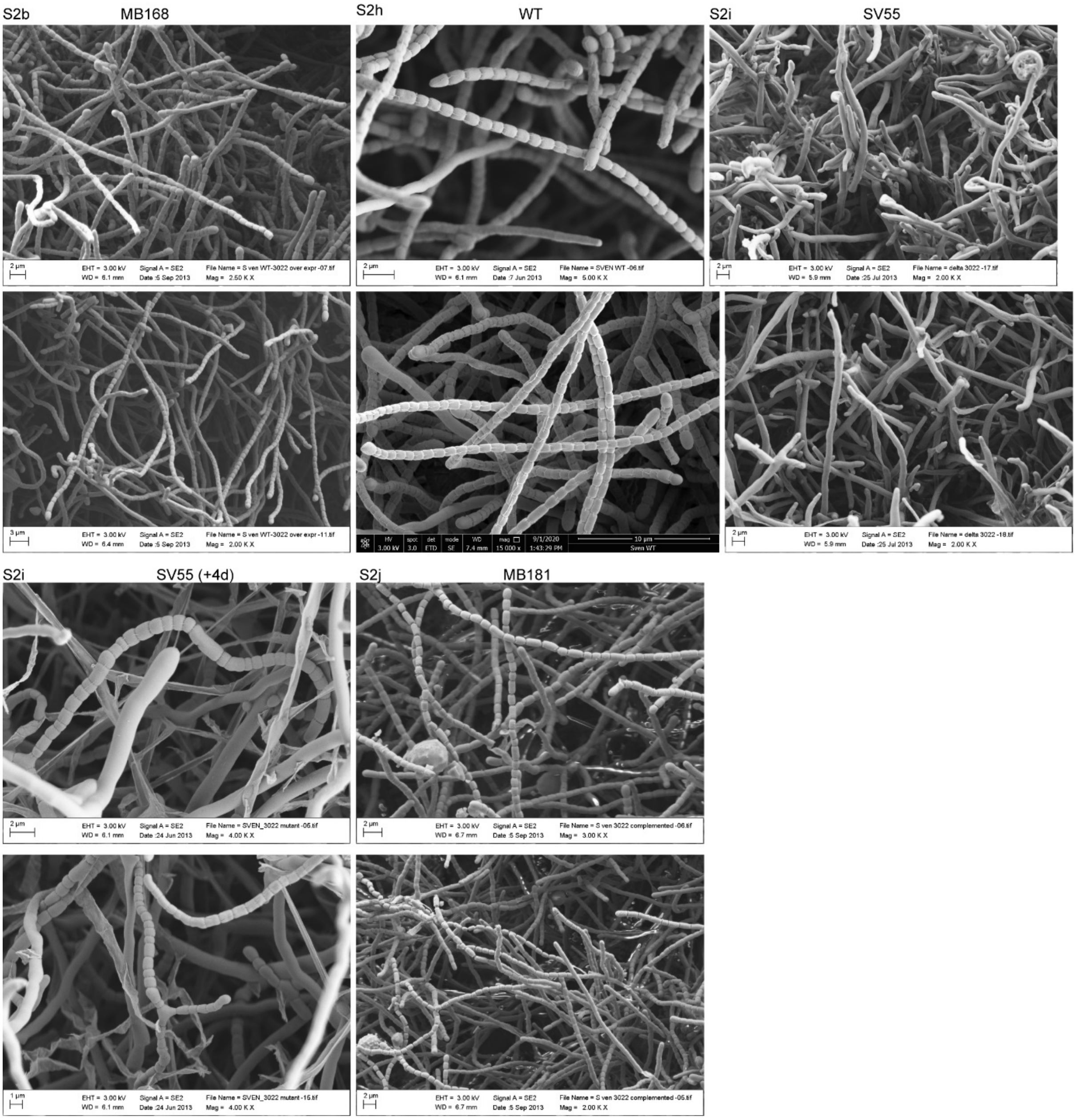

Supplementary Figure 12: Full uncropped SEM image and additional SEMs corresponding to Supplementary Figure 2. Note that the first SEM images corresponding to Supplementary Figure 2b was reflected vertically and the first image showing SEM S2j was rotated by 90°.

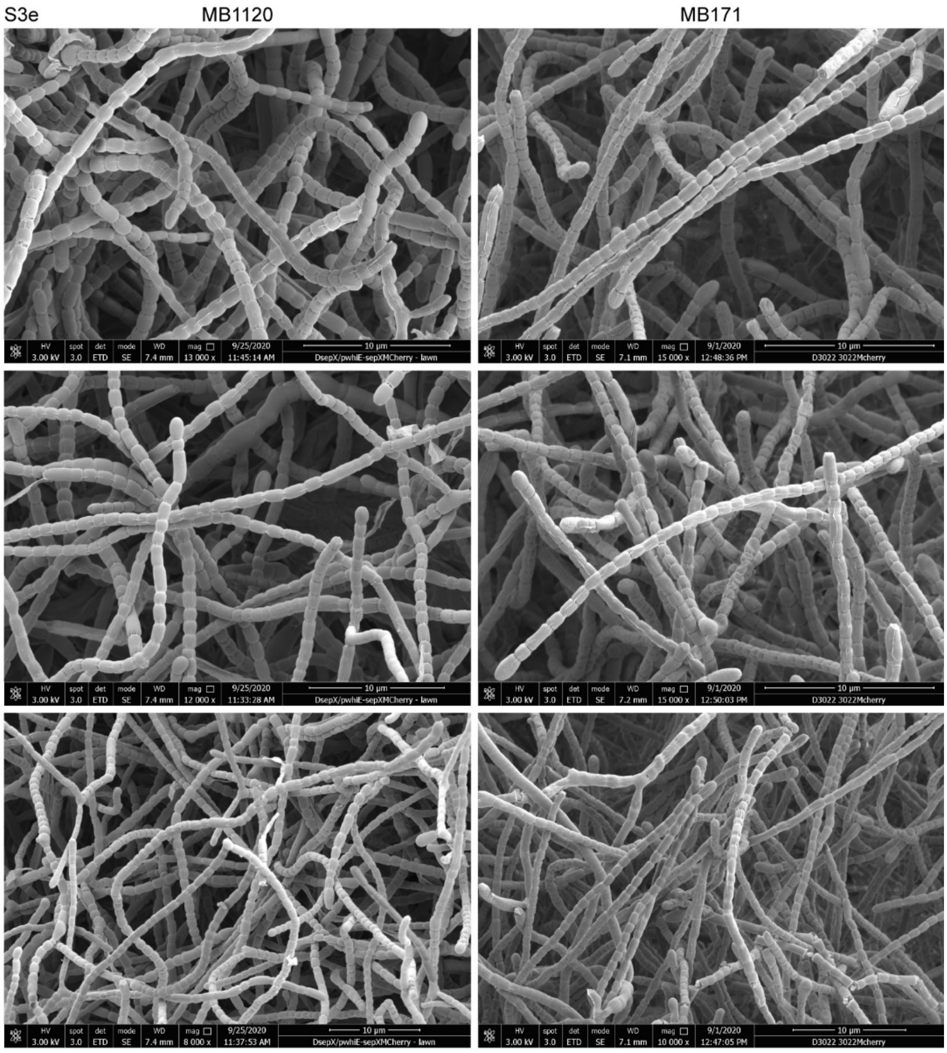

Supplementary Figure 13: Full uncropped SEM images and additional SEMs corresponding to Supplementary Figure 3. Note that the first SEM images corresponding to Supplementary Figure 3e was reflected vertically.

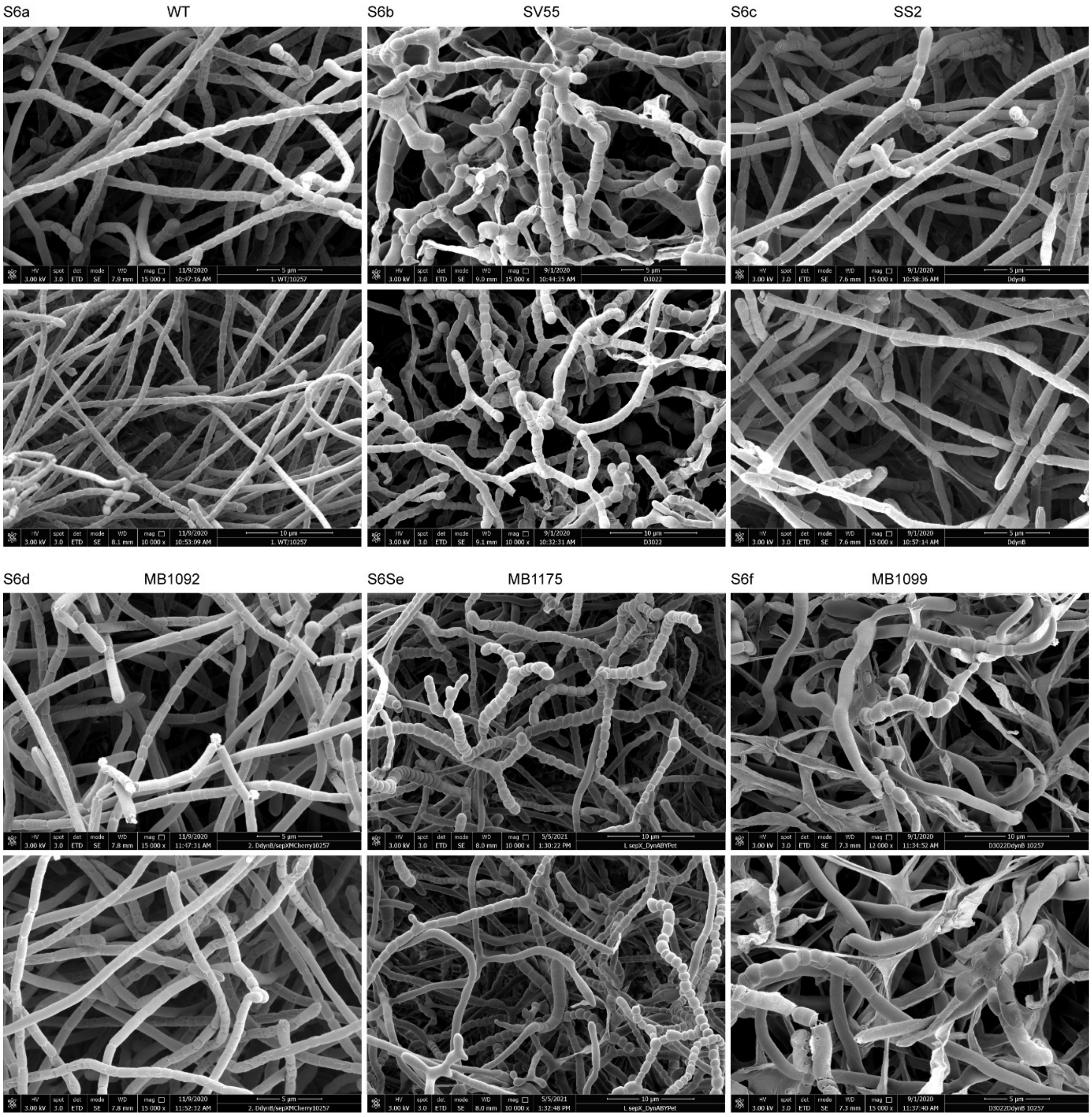

Supplementary Figure 14: Full uncropped SEM images corresponding to Supplementary Figure 6. For each strain, an additional representative SEM image is shown below.

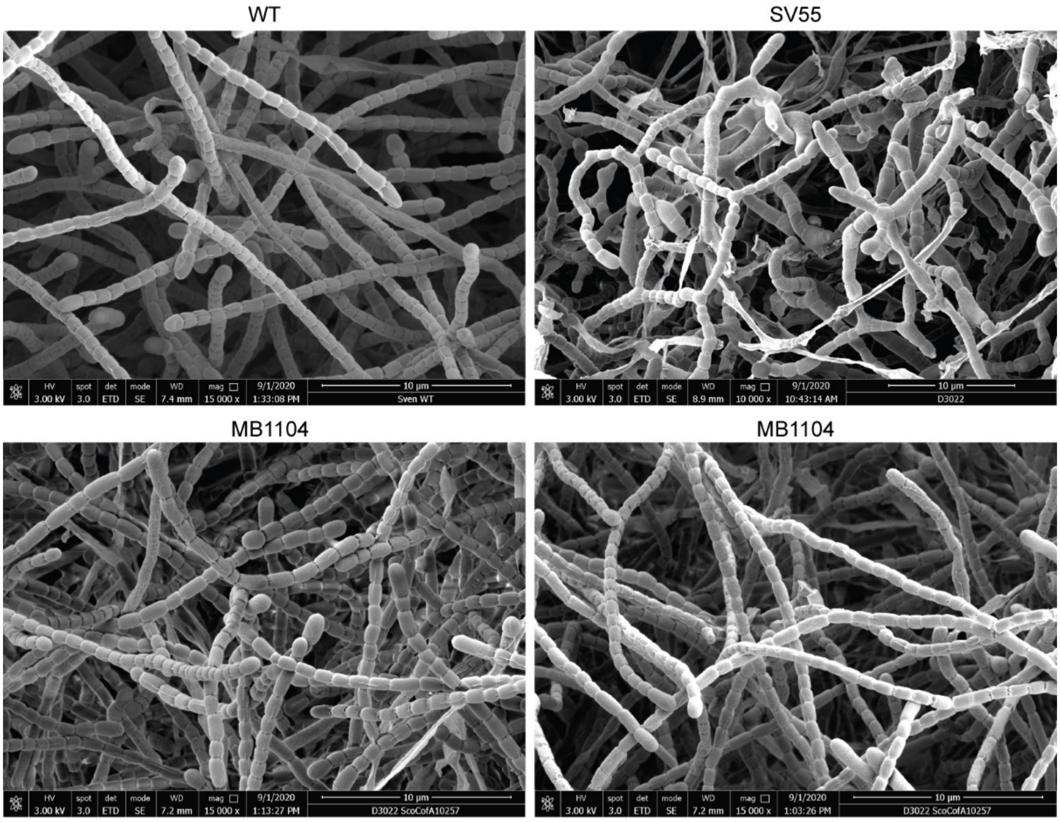

Supplementary Figure 15: Full uncropped SEM images corresponding to Supplementary Figure 8. For MB1104, an additional representative SEM image is shown.

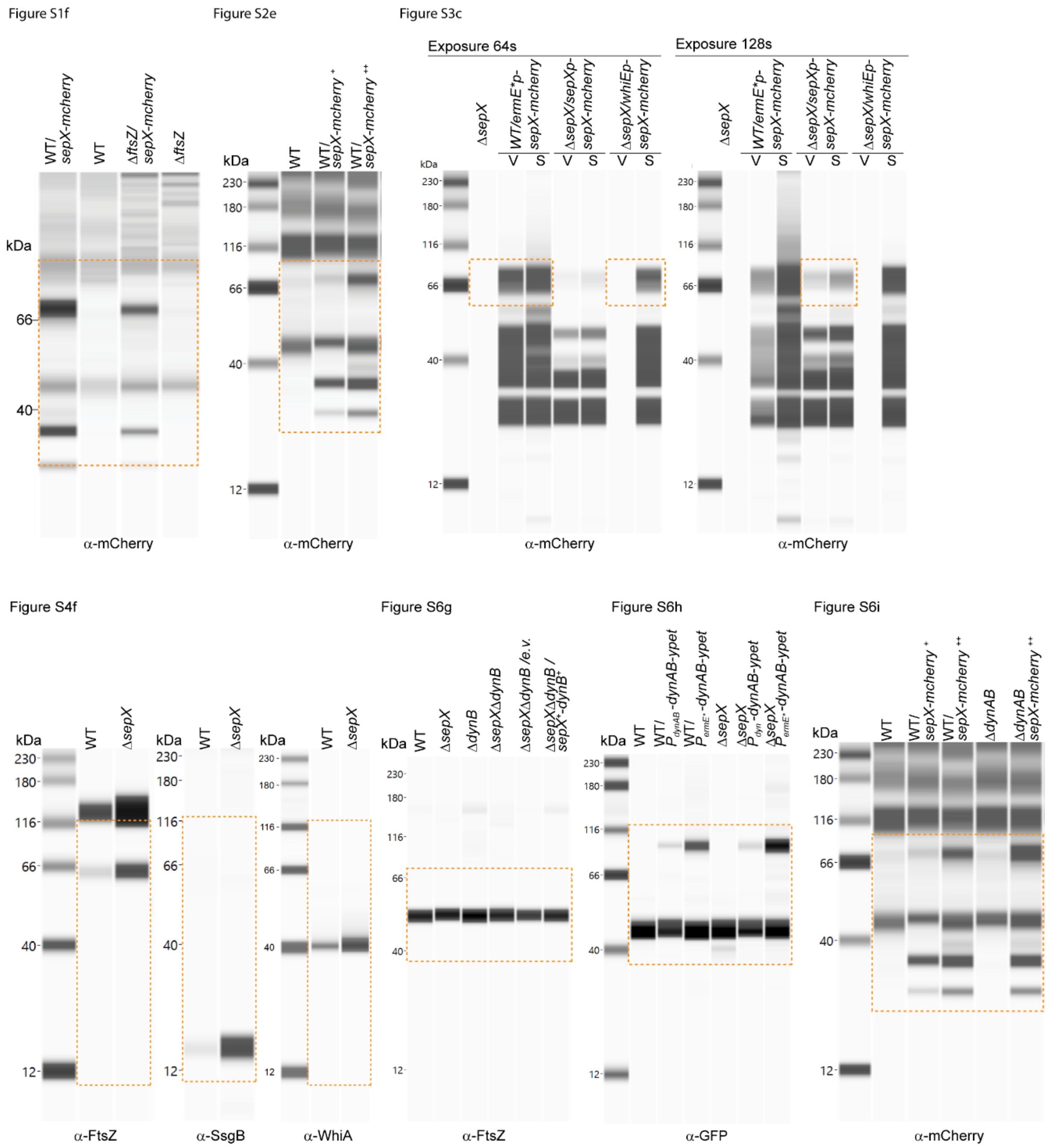

Supplementary Figure 16: Full uncropped virtual Western blots of all the analysis shown in this work. The orange boxes correspond to the crops used in the figures.

Supplementary Table 1: Strains used in this work.

**Strain**	**Details**	**Source**	**Reference**
S. venezuelae			
NRRL B-65442	Wild type (WT)	Laboratory Strain	[2]
SV7	Δ*whiA*::*apr*		[3]
SV11	Δ*whiB*::*apr*		[4]
SV55	Δ*sepX*::*apr*	Chromosomal *sepX* locus was replaced by *apra-oriT* by "redirect" PCR	This work
SV57	Δ*sepX*::*hyg ΔdynB::apr*	Chromosomal *sepX* locus was replaced by *hyg-oriT* by "Redirect" in SS2 background	This work
DU669	*ΔftsZ::apr*		[5]
LUV001	*ΔdynAB::apr*		[6]
LUV015	WT $attB_{\Phi C31}$::P_{ftsZ}-*ftsZ-ypet*		[6]
SS2	*ΔdynB::apr*		[6]
SS4	WT $attB_{\Phi BT1}$::pIJ10257	pIJ10257 integrated into the ΦBT1-attachment site of *S. venezuelae*	[6]
SS10	*ΔdynAB::apr* $attB_{\Phi BT1}$::pIJ10257	pIJ10257 integrated into the ΦBT1-attachment site of LUV001	[6]
SS12	WT $attB_{\Phi BT1}$::P_{ftsZ}-*ftsZ-ypet*	pSS5 integrated into the ΦBT1-attachment site of *S. venezuelae*	[6]
SS92	WT $attB_{\phi BT1}$::P_{dyn}-*dynA-dynB-ypet*	pSS89 integrated into the ΦBT1-attachment site of *S. venezuelae*	This work
SS155	*ΔsepX::apr* $attB_{\phi BT1}$::P_{dyn}-*dynA-dynB-ypet*	pSS89 integrated into the ΦBT1-attachment site of SV55	This work
SS414	WT $attB_{\Phi BT1}$::P_{ermE*}-*sepF-3xFLAG*	pSS559 integrated into the ΦBT1-attachment site of *S. venezuelae*	This work
MB127	WT $attB_{\Phi BT1}$::P_{ermE*}-*ftsZ*	pSS3 integrated into the ΦBT1-attachment site of *S. venezuelae*	This work
MB168	WT $attB_{\Phi BT1}$::*ermE*-sepX*	pMB156 integrated into the ΦBT1-attachment site of *S. venezuelae*	This work
MB170	*WT* $attB_{\Phi BT1}$:: P_{sepX}-*sepX-mcherry*	pMB192 integrated into the ΦBT1-attachment site of *S. venezuelae*	This work
MB171	*ΔsepX::apr* $attB_{\Phi BT1}$:: P_{sepX}-*sepX-mcherry*	pMB192 integrated into the ΦBT1-attachment site of SV55	This work
MB180	Δ*sepX::apr* $attB_{\Phi BT1}$::P_{ftsZ}-*ftsZ-ypet*	pSS5 integrated into the ΦBT1-attachment site of SV55	This work
MB181	Δ*sepX::apr* $attB_{\Phi BT1}$::P_{sepX}-*sepX*	pMB182 integrated into the ΦBT1-attachment site of SV55	This work
MB182	Δ*sepX::apr* $attB_{\Phi BT1}$::P_{ermE*}-*sepX*	pMB156 integrated into the ΦBT1-attachment site of SV55	This work
MB192	Δ*sepX::apr* $attB_{\Phi BT1}$::pMS82	pMS82 integrated into the ΦBT1-attachment site of SV55	This work
MB256	WT $attB_{\Phi BT1}$::P_{sepX}-*sepX-mcherry* $attB_{\Phi C31}$::P_{ftsZ}-*ftsZ-ypet*	pMB192 integrated into the ΦBT1-attachment site and pKF351 integrated into the ΦC31-attachment site of *S. venezuelae*	This work

Strain	Details	Source	Reference
MB745	Δ*sepX::apr* $attB_{\Phi BT1}$::P_{sepX}-*sepX*	pMB556 integrated into the ΦBT1-attachment site of SV55	This work
MB839	Δ*ftsZ::apr* $attB_{\Phi BT1}$::pIJ10257	pIJ10257 integrated into the ΦBT1-attachment site of DU669	This work
MB862	Δ*sepX::apr* $attB_{\Phi BT1}$::pIJ10257	pIJ10257 integrated into the ΦBT1-attachment site of SV55	This work
MB942	Δ*sepX::apr* $attB_{\Phi BT1}$::P_{sepX}-*sepX-3xFLAG*/P_{dynAB}-*dynA-dynB-ypet*	pMB703 integrated into the ΦBT1-attachment site of SV55	This work
MB1104	Δ*sepX::apr* $attB_{\Phi BT1}$::P_{ermE*}-*Sco-sepX*	pMB741 integrated into the ΦBT1-attachment site of SV55	This work
MB1082	Δ*ftsZ::apr* $attB_{\Phi BT1}$::P_{ermE*}-*sepX-mcherry*	pMB748 integrated into the ΦBT1-attachment site of DU669	This work
MB1092	Δ*dynAB::apr* $attB_{\Phi BT1}$:: P_{ermE*}-*sepX-mcherry*	pMB748 integrated into the ΦBT1-attachment site of LUV001	This work
MB1099	Δ*sepX::hyg* Δ*dynB::apr* $attB_{\Phi BT1}$::*pIJ10257*	pMB743 integrated into the ΦBT1-attachment site of SV57	This work
MB1101	Δ*sepX::hyg* Δ*dynB::apr* $attB_{\Phi BT1}$:: *ermE*-dynB*	pMB745 integrated into the ΦBT1-attachment site of SV57	This work
MB1102	Δ*sepX::hyg* Δ*dynB::apr attBΦBT1:: ermE*-sepX*	pMB744 integrated into the ΦBT1-attachment site of SV57	This work
MB1103	Δ*sepX::hyg* Δ*dynB::apr* $attB_{\Phi BT1}$:: P_{ermE*}-*dynB*; P_{sepX}-*sepX*	pMB746 integrated into the ΦBT1-attachment site of SV57	This work
MB1111	Δ*sepX::hyg* Δ*dynB::apr* $attB_{\Phi BT1}$::*pKF351*	pKF351 integrated into the ΦBT1-attachment site of SV57	This work
MB1120	Δ*sepX::apr* $attB_{\Phi BT1}$:: P_{whiE}-*sepX-mcherry*	pSS601 integrated into the ΦBT1-attachment site of SV55	This work
MB1124	WT $attB_{\Phi BT1}$::P_{ermE*}-*sepX-mcherry*	pMB748 integrated into the ΦBT1-attachment site of *S. venezuelae*	This work
MB1175	Δ*sepX::apr* $attB_{\phi BT1}$::P_{ermE*}-*dynA-dynB-ypet*	pSS134 integrated into the ΦBT1-attachment site of SV55	This work
MB1180	WT $attB_{\phi BT1}$:: P_{ermE*}-*dynA-dynB-ypet*	pSS134 integrated into the ΦBT1-attachment site of *S. venezuelae*	This work
MB1270	Δ*dynAB*::*apr* $attB_{\Phi BT1}$::P_{sepX}-*sepX-mcherry*	pMB192 integrated into the ΦBT1-attachment site of LUV001	This work
E.coli			
ET12567(pUZ8002)	F^- *dam13::Tn9 dcm6 hsdM hsdR recF143::Tn10 galK2 galT22 ara-14 lacY1 xyl-5leuB6 thi-1 tonA31 rpsL hisG4 tsx-78 mtl-1 glnV44*	ET12567 containing helper plasmid pUZ8002	[7]
BW25113	Δ(*araD-araB*)*567* Δ *lacZ4787*(::*rrnB-4*) *lacIp-4000*($lacI^{Q}$), l-*rpoS369*(*Am*) *rph-1* Δ(*rhaD-rhaB*)*568 hsdR514*	BW25113 containing λ RED recombination plasmid	[8]
BTH101	F^- *cya-99 araD139 galE15 galK16 rpsL1* (Str^{R}) *hsdR2 mcrA1 mcrB1*	Bacterial two-hybrid host strain	[9]

Supplementary Table 2: Plasmids used in this work.

Plasmid	Details	Construction	Reference
pIJ773	pBluescript KS (+) containing the apramycin resistance gene *apr* and *oriT* of plasmid RP4, flanked by FRT sites (Apr^R). Used as template for the amplification of the *apr oriT* cassette for 'Redirect' PCR-targeting		[10]
pIJ10700	pBluescript KS (+) containing the hygromycin resistance gene *hyg* and *oriT* of plasmid RP4, flanked by FRT sites (Apr^R). Used as template for the amplification of the *hyg oriT* cassette for 'Redirect' PCR-targeting		[9]
pIJ790	Modified λ RED recombination plasmid *[oriR101] [repA101*(ts)*] araBp-gam-bet-exo* (Cam^R)		[9]
pIJ10754	pUC19-derivative used to generate 3xFLAG gene fusions (Amp^R)		[6]
pIJ10750	pMS82 with an extended Multiple Cloning Site, (Hyg^R)		[5]
pIJ10770	Modified pIJ10750 lacking an intrinsic apramycin promoter upstream of the extended multiple cloning site (Hyg^R)		[5]
pIJ10257	Plasmid cloning vector for the conjugal transfer of DNA (under control of the *ermE** constitutive promoter) from *E. coli* to *Streptomyces spp.* Integrates specifically at the ΦBT1 attachment site (Hyg^R)		[11]
pIJ6772	pKT25 carrying *rsbN* (Kan^R)		[12]
pIJ6769	pUT18C carrying *bldN* (Amp^R)		[11]
pMS82	Plasmid cloning vector for the conjugal transfer of DNA from *E. coli* to *Streptomyces* spp. Integrates site specifically at the ΦBT1 attachment site (Hyg^R)		[13]
pTB145	Plasmid for overexpression of His6-Upl1 protease (Amp^R)		[14]

Plasmid	Details	Construction	Reference
pTB146	Plasmid for overexpression of proteins with N-terminal His$_6$-SUMO fusion (Amp^R)		[14]
pKF280	Vector for the construction of gene fusions to *ypet*, based on pIJ6902 but with the *tipA* promoter removed. (Apr^R)		[15]
pKF351	Derivative of pKF280 encoding the FtsZ-YPet fusion (Apr^R)		[6]
pKT25	Two-hybrid plasmid, N-terminal *cyaAT25* fusion (Kan^R)		[9]
pKNT25	Two-hybrid plasmid, C-terminal *cyaAT25* fusion (Kan^R)		[8]
pUT18	Two-hybrid plasmid, C-terminal *cyaAT18* fusion (Amp^R)		[8]
pUT18C	Two-hybrid plasmid, N-terminal *cyaAT18* fusion (Amp^R)		[8]
pSS3	pIJ10257 carrying *ftsZ* (Hyg^R)		[6]
pSS5	pIJ10750 carrying *pftsZ-ftsZ-ypet (*Hyg^R)		[6]
pSS23	pKT25 carrying *dynA* (Kan^R)	*dynA* (*vnz_12110*) was PCR amplified using primers ss65/66 and inserted into pKT25 via restriction cloning using EcoRI/BamHI.	This work
pSS64	pIJ10257 carrying *dynB* (Hyg^R)	*dynB (vnz_12105)* was PCR amplified using primers ss21/97 and inserted into pIJ10257 via restriction cloning using NdeI/HindIII.	This work
pSS89	pKF227 carrying P_{dynAB}*-dynA-dynB-ypet* (Hyg^R)		[6]
pSS105	pKT25 carrying *dynB* (Kan^R)		[6]
pSS115	pUT18C carrying *dynB* (Amp^R)		[6]
pSS125	pKT25 carrying *dynB*Δ468-517 (Kan^R)	*dynB▯TM* (ΔAA468-517) was generated by amplifying the two *dynB* fragments using primers ss69/190 and ss70/189, followed by restriction digestion with EcoRI/Asp718 and Asp718/BamHI. Both fragments were ligated between the EcoRI-BamHI site of pKT25.	This work

**Plasmid**	**Details**	**Construction**	**Reference**
pSS133	pIJ10257 carrying *dynB-3xFLAG* (Hyg^R)	*dynB* was amplified using primer ss105/106 and first cloned into pIJ10754 between the KpnI-XhoI site. The *dynB-3xFLAG* fragment was then amplified with primer ss209/21 and subcloned into pIJ10257 between the NdeI-HindIII site.	This work
pSS134	pIJ10257 carrying *dynA-dynB-ypet* (Hyg^R)		[6]
pSS172	pIJ10750 carrying *mcherry* (Hyg^R)		[6]
pSS196		*sepF (vnz_08505)* was PCR amplified using primer ss336/337 and inserted into pSS172 via restriction cloning using HindIII/AvrII.	This work
pSS220	pKT25 carrying *sepF2* (Kan^R)		[6]
pSS221	pKT25 carrying *sepF3* (Kan^R)		[6]
pSS222	pKT25 carrying *sepF* (Kan^R)		[6]
pSS440	pTB146 carrying ssgB ($Carb^R$)	*ssgB (vnz_05545)* was PCR amplified with primer ss889/ss890, followed by gibson assembly into pTB146 cut with BpsQI	This work
pSS559	pIJ10257 carrying *sepF-3xFLAG* (Hyg^R)	*sepF (vnz_08505)* was PCR amplified from pSS196 with primer ss418/ss5, followed by restriction digestion with NdeI/XhoI; *3xFLAG* fragment was isolated from pSS133 cut with NcoI/XhoI; pIJ10257 was cut with NcoI/NdeI; all three fragments were assembled by ligation.	This work
pSS601	pMB748 modified so that *sepX-mcherry* under the control of P_{whiE} (Hyg^R)	*whiE* promoter region was PCR amplified with primer ss1372/ss1373 and inserted via Gibson Assembly into pMB748 cut with Bsu36I and NdeI to remove *ermE** promoter.	This work
pMB156	pIJ10257 carrying *sepX* (Hyg^R)	*sepX* was PCR amplified using primers mb331/mb332 and inserted into pIJ10257 via restriction cloning using NdeI/HindIII.	This work

Plasmid	Details	Construction	Reference
pMB174	pKT25 carrying *ssgB* (Kan^R)	*ssgB* was PCR amplified using primers mb364/mb365 and inserted into pKT25 via restriction cloning using BamHI/KpnI.	This work
pMB177	pKNT25 carrying *sepX* (Kan^R)	*sepX* was PCR amplified using primers mb366/mb367 and inserted into pKNT25 via restriction cloning using XbaI/BamHI.	This work
pMB180	pUT18 carrying *sepX* (Amp^R)	*sepX* was PCR amplified using primers mb366/mb367 and inserted into pUT18 via restriction cloning using XbaI/BamHI.	This work
pMB182	pIJ10750 carrying P_{sepX}-*sepX* (Hyg^R)	P_{sepX}-*sepX* was PCR amplified using primers mb210/mb340 and inserted into pIJ10750 via restriction cloning using HindIII/AvrII.	This work
pMB192	pIJ10750 carrying P_{sepX}-*sepX-mcherry* (Hyg^R)	Two-step amplification using mb210/mb382 and mb384/mb385 in the first step. Templates from first step amplified using mb210 and mb385 to generate P_{sepX}-*sepX -mcherry* fragment which was inserted into pIJ10750 via restriction cloning using HindIII/AvrII.	This work
pMB555	pIJ10770 carrying P_{sepX}-*sepX-FLAG* (Hyg^R)	Two-step amplification using mb373/mb377 and mb378/mb374 in the first step. Templates from first step amplified using mb373 and mb374 to generate P_{sepX}-*sepX-FLAG* fragment which was inserted into pIJ10770 via restriction cloning using HindIII/AvrII.	This work
pMB556	pIJ10770 carrying P_{sepX}-*sepX* (Hyg^R)	P_{sepX}-*sepX* was PCR amplified using mb1433/mb1434 and pMB182 as a template and cloned into EcoRV-cut pIJ10770 using Gibson Assembly.	This work
pMB703	pSS89 carrying P_{sepX}-*sepX-3xFLAG* (Hyg^R)	P_{sepX}-*sepX-3xFLAG* was PCR amplified using mb1150/mb1151 and pMB555 as a template and cloned into EcoRV-cut pSS89 using Gibson Assembly.	This work
pMB741	pIJ10257 carrying *S. coelicolor sepX*	*Sco3177* was PCR amplified using mb1437/mb1438 and cosmid stE87 as a template and inserted into pIJ10257 via restriction cloning using NdeI/HindIII.	This work

Plasmid	Details	Construction	Reference
pMB744	pMB156 carrying additional *thio*^{R}	Thiostrepton resistance cassette was amplified from pKF351 with primer mb1451/mb1452 followed by Gibson Assembly into EcoRV-cut pMB156.	This work
pMB745	pSS64 carrying additional *thio*^{R}	Thiostrepton resistance cassette was amplified from pKF351 with primer mb1451/mb1452 followed by Gibson Assembly into EcoRV-cut pSS64.	This work
pMB746	pMB745 carrying P_{sepX}-*sepX*	P_{sepX}-*sepX* was amplified using primers mb1453/mb1454 and pMB192 as template and assembled into SpeI-cut pMB745.	This work
pMB748	pIJ10257 carrying *sepX-mcherry*	*sepX-mcherry* was PCR amplified using primers mb331/mb1448 and pMB192 as template and inserted into pIJ10257 via restriction cloning using NdeI/HindIII.	This work

Supplementary Table 3: Primer used in this work.

Primer	Sequence
mb210	GGCGAAGCTTCCCGCTACCTGCACATCG
mb266	TGCCGTCCCGCGAACCCCTCTACCGTGTGAGGCGTGAGCATTCCGGGGATCCGTCGACC
mb267	CAGCCGGGGGCCGTCGCGGCGCCCCCGGCGGGGTCATTCTGTAGGCTGGAGCTGCTTC
mb331	GGAATTCCATATGAGCAGCAGCGGCCTCA
mb332	CCCAAGCTTTCATTCGTTGGCCGCCCG
mb340	GCTGCCTAGGTCATTCGTTGGCCGCCCG
mb364	CTGAGGATCCCATGAACACCACGGTCAGCTG
mb365	CCGGTACCCGGCTGTCGGCCAGGATGTG
mb366	GCTCTAGAGGTGAGCAGCAGCGGCCTC
mb367	CGCGGATCCTCTTCGTTGGCCGCCCGGG
mb373	GGCGAAGCTTCCCGCTACCTGCACATCGACG
mb374	GCTGCCTAGGCCACAGGCCCTTGCGACGTG
mb377	TCGATGTCGTGGTCCTTGTAGTCGCCGTCGTGGTCCTTGTAGTCTTCGTTGGCCGCCCGGGG
mb378	CGACTACAAGGACCACGACATCGACTACAAGGACGATGACGACAAGTGACCCCGCCGGGGGCG
mb382	CTTGGAGACTTCGTTGGCCGCCCGGGG
mb384	GCCAACGAAGTCTCCAAGGGCGAGGAG
mb385	GCTGCCTAGGTCACTTGTACAGCTCGTCCATG
mb1150	GGAGCGCGGCCGCGCGCGATCCCGCTACCTGCACATCG
mb1151	GACATGATTACGAATTCGATCCACAGGCCCTTGCGACG
mb1431	GTGCCGGAGGGGCTGCTG
mb1432	GCATCCCGACCGGCTCAGG
mb1433	GCGGCCGCGCGCGATCCCGCTACCTGCACATCG
mb1434	ACATGATTACGAATTCGATTCATTCGTTGGCCGCCCG
mb1437	GGGAATTCCATATGAGCAGCAGCGGCCTCATCT
mb1438	CCCAAGCTTCTACTCGTTGGCCGCGCGG
mb1448	CCCAAGCTTTCACTTGTACAGCTCGTCCATGCCG

Primer	Sequence
mb1451	ACCATAGCGGGCAGGGAGCGGATCCGCGGCCGCGCGCGATATCGCTCATGAGCGGAGAACGAGATGACGTTGG AGGGGCAAGGTCGCG
mb1452	TTCACACAGGAAACAGCTATGACATGATTACGAATTCGATATCCTTATCGGTTGGCCGCGAGATTCCT
mb1453	TCGGCCCCTTTTTTGGCCTTGAAATCGTTAGTTAGGCTAACCCGCTACCTGCACATCGACG
mb1454	GCGGATCCGCTCCCTGCCCGCTATGGTGACGAAGGAACTATCATTCGTTGGCCGCCCGG
ss5	TGGCCGTTCACGGAGCCCTC
ss21	ATTAATTCATATGGTGGATGTGGACGTGGAAGCG
ss65	TTAAGGATCCCATGGTGACCTTGGACGAACGGCC
ss66	AATGAATTCTCACTTCTCCTTCTGCAGTACGGAGAGC
ss69	AATTAAGGATCCCATGGTGGATGTGGACGTGGAAGCG
ss70	AATTAATGAATTCCTACCTGCCCCGTACCCCCGTC
ss97	TTAATTCCTAGGCTACCTGCCCCGTACCCCCGTCC
ss105	ATCGGGGTACCGTGGTGGATGTGGACGTGGAAGCG
ss106	ATCCGCTCGAGCCTGCCCCGTACCCCCGTCCGCG
ss189	ATCGGGGTACCCCGGTCCTCGTGATGGTCGGCG
ss190	ATCGGGGTACCGACCGTCAGCTCGTCGAGCGCCTC
ss209	GAGCGGATAACAATTTCACACAGG
ss336	ATTAAAGCTTAGCTCGGCTGCCTCCTGCAGGTC
ss337	TTAATTCCTAGGGCTCTGGTTGAAGAATCCGCCCTCTG
ss418	AATTAATTCATATGGCCGGCGCGATGCGCAAG
ss889	TGAGGCTCACAGAGAACAGATTGGTGGTAGAAGAGCAATGAACACCACGGTCAGCTG
ss890	TTGTCGACGGAGCTCTGCTCTTCTATCAGCTGTCGGCCAGGATGT
ss1372	CCTCGCCTCTGACCCCTGACCCCGTCAGCCTCCCCGGCCGAGTCGAATCGG
ss1373	TGACTGCGTAGATGAGGCCGCTGCTGCTCATATGGCACACCTCTTCTCTTCTCGGTGGGGGGTGGGG

Supplementary References

1. Al-Bassam, M. M., Bibb, M. J., Bush, M. J., Chandra, G. & Buttner, M. J. Response regulator heterodimer formation controls a key stage in *Streptomyces* development. *PLOS Genetics* **10**, e1004554 (2014).
2. Gomez-Escribano, J. P. *et al. Streptomyces venezuelae* NRRL B-65442: genome sequence of a model strain used to study morphological differentiation in filamentous actinobacteria. *J Ind Microbiol Biotechnol* kuab035 (2021) doi:10.1093/jimb/kuab035.
3. Bush, M. J., Bibb, M. J., Chandra, G., Findlay, K. C. & Buttner, M. J. Genes required for aerial growth, dell division, and chromosome segregation are targets of WhiA before sporulation in *Streptomyces venezuelae*. *mBio* **4**, e00684-13 (2013).
4. Bush, M. J., Chandra, G., Bibb, M. J., Findlay, K. C. & Buttner, M. J. Genome-wide chromatin immunoprecipitation sequencing analysis shows that WhiB Is a transcription factor that cocontrols its regulon with WhiA to initiate developmental dell division in *Streptomyces*. *mBio* **7**, e00523-16, /mbio/7/2/e00523-16.atom (2016).
5. Santos-Beneit, F., Roberts, D. M., Cantlay, S., McCormick, J. R. & Errington, J. A mechanism for FtsZ-independent proliferation in *Streptomyces*. *Nat Commun* **8**, 1378 (2017).
6. Schlimpert, S. *et al.* Two dynamin-like proteins stabilize FtsZ rings during *Streptomyces* sporulation. *PNAS* **114**, E6176–E6183 (2017).

7. Paget, M. S. B., Chamberlin, L., Atrih, A., Foster, S. J. & Buttner, M. J. Evidence that the extracytoplasmic function sigma factor σE is required for normal cell wall structure in *Streptomyces coelicolor* A3(2). *J. Bacteriol.* **181**, 8 (1999).
8. Datsenko, K. A. & Wanner, B. L. One-step inactivation of chromosomal genes in *Escherichia coli* K-12 using PCR products. *PNAS* **97**, 6640–6645 (2000).
9. Karimova, G., Pidoux, J., Ullmann, A. & Ladant, D. A bacterial two-hybrid system based on a reconstituted signal transduction pathway. *PNAS* **95**, 5752–5756 (1998).
10. Gust, B., Challis, G. L., Fowler, K., Kieser, T. & Chater, K. F. PCR-targeted *Streptomyces* gene replacement identifies a protein domain needed for biosynthesis of the sesquiterpene soil odor geosmin. *PNAS* **100**, 1541–1546 (2003).
11. Hong, H.-J., Hutchings, M. I., Hill, L. M. & Buttner, M. J. The role of the novel Fem protein VanK in vancomycin resistance in *Streptomyces coelicolor*. *J. Biol. Chem.* **280**, 13055–13061 (2005).
12. Bibb, M. J., Domonkos, Á., Chandra, G. & Buttner, M. J. Expression of the chaplin and rodlin hydrophobic sheath proteins in *Streptomyces venezuelae* is controlled by σBldN and a cognate anti-sigma factor, RsbN. *Molecular Microbiology* **84**, 1033–1049 (2012).
13. Gregory, M. A., Till, R. & Smith, M. C. M. Integration site for *Streptomyces* phage phiBT1 and development of site-specific integrating vectors. *J. Bacteriol.* **185**, 5320–5323 (2003).
14. Bendezú, F. O., Hale, C. A., Bernhardt, T. G. & de Boer, P. A. J. RodZ (YfgA) is required for proper assembly of the MreB actin cytoskeleton and cell shape in *E. coli*. *EMBO J.* **28**, 193–204 (2009).
15. Donczew, M. *et al.* ParA and ParB coordinate chromosome segregation with cell elongation and division during *Streptomyces* sporulation. *Open Biol* **6**, 150263 (2016).
