# Supplementary material for: Hyphal compartmentalization and sporulation in Streptomyces require the conserved cell division protein SepX
Source: Nat Commun. 2022 Jan 10;13:71. doi: 10.1038/s41467-021-27638-1 (PMC8748795; doi:10.1038/s41467-021-27638-1)
Supplement: Supplementary file 3 — Description of Additional Supplementary Files [file 41467_2021_27638_MOESM3_ESM.pdf]

Description of Additional Supplementary Files

File Name: Supplementary Movie 1
Description: Fluorescence time-lapse movie showing FtsZ-YPet and SepX-mCherry co-localization in wild-type *S. venezuelae* (MB256) during growth and sporulation.

File Name: Supplementary Movie 2
Description: Fluorescence time-lapse movie showing FtsZ-YPet localization in wild-type *S. venezuelae* (SS12) during growth and sporulation.

File Name: Supplementary Movie 3
Description: Fluorescence time-lapse movie showing FtsZ-YPet localization in the Δ*sepX* mutant (MB180) during growth and sporulation.

File Name: Supplementary Movie 4
Description: Fluorescence time-lapse movie showing FtsZ-YPet localization in the Δ*sepX*Δ*dynB* double mutant (MB1111) during growth and sporulation.
