# Supplementary material for: Hyphal compartmentalization and sporulation in Streptomyces require the conserved cell division protein SepX
Source: Nat Commun. 2022 Jan 10;13:71. doi: 10.1038/s41467-021-27638-1 (PMC8748795; doi:10.1038/s41467-021-27638-1)
Supplement: Supplementary file 8 — Reporting Summary [file 41467_2021_27638_MOESM8_ESM.pdf]

nature portfolio

Corresponding author(s): Susan Schlimpert
Last updated by author(s): Oct 28, 2021

Reporting Summary

Nature Portfolio wishes to improve the reproducibility of the work that we publish. This form provides structure for consistency and transparency in reporting. For further information on Nature Portfolio policies, see our Editorial Policies and the Editorial Policy Checklist.

Statistics

For all statistical analyses, confirm that the following items are present in the figure legend, table legend, main text, or Methods section.

n/a	Confirmed	
☐	☒	The exact sample size (*n*) for each experimental group/condition, given as a discrete number and unit of measurement
☐	☒	A statement on whether measurements were taken from distinct samples or whether the same sample was measured repeatedly
☐	☒	The statistical test(s) used AND whether they are one- or two-sided<br>*Only common tests should be described solely by name; describe more complex techniques in the Methods section.*
☒	☐	A description of all covariates tested
☒	☐	A description of any assumptions or corrections, such as tests of normality and adjustment for multiple comparisons
☐	☒	A full description of the statistical parameters including central tendency (e.g. means) or other basic estimates (e.g. regression coefficient) AND variation (e.g. standard deviation) or associated estimates of uncertainty (e.g. confidence intervals)
☐	☒	For null hypothesis testing, the test statistic (e.g. *F*, *t*, *r*) with confidence intervals, effect sizes, degrees of freedom and *P* value noted<br>*Give P values as exact values whenever suitable.*
☒	☐	For Bayesian analysis, information on the choice of priors and Markov chain Monte Carlo settings
☒	☐	For hierarchical and complex designs, identification of the appropriate level for tests and full reporting of outcomes
☒	☐	Estimates of effect sizes (e.g. Cohen's *d*, Pearson's *r*), indicating how they were calculated

Our web collection on statistics for biologists contains articles on many of the points above.

Software and code

Policy information about availability of computer code

Data collection	Zeiss ZenBlue Version 2.3, Compass for SW Version 6.0.0, Multisizer 4 Version 4.01, Mascot v.2.3
Data analysis	GraphPad Prism 9, Microsoft Excel 2013, Compass for SW Version 6.0.0, Fiji (ImageJ 1.53c), Scaffold4 Software for MS/MS Proteomics Version 4.0.7, Adobe Illustrator CS6.

For manuscripts utilizing custom algorithms or software that are central to the research but not yet described in published literature, software must be made available to editors and reviewers. We strongly encourage code deposition in a community repository (e.g. GitHub). See the Nature Portfolio guidelines for submitting code & software for further information.

Data

Policy information about availability of data

All manuscripts must include a data availability statement. This statement should provide the following information, where applicable:

- Accession codes, unique identifiers, or web links for publicly available datasets
- A description of any restrictions on data availability
- For clinical datasets or third party data, please ensure that the statement adheres to our policy

Additional data is available on request from the corresponding author. Source Data is provided with this manuscript.

Field-specific reporting

Please select the one below that is the best fit for your research. If you are not sure, read the appropriate sections before making your selection.

☒ Life sciences ☐ Behavioural & social sciences ☐ Ecological, evolutionary & environmental sciences

For a reference copy of the document with all sections, see nature.com/documents/nr-reporting-summary-flat.pdf

Life sciences study design

All studies must disclose on these points even when the disclosure is negative.

Sample size	No statistical test were used to determine sample size. Sample sizes are based on established protocols and the potential to provided reliable and representative data, or was determined by the experimental conditions, e.g. automated cell counting.
Data exclusions	Coulter counter data presented in Fig. 2d, Supplementary Fig. 3f: for clarity, data points with a volume larger than 5 micro-meter^3 are not shown. This was based on the visual inspection of the individual spore stocks showing the presence of larger fragments that are likely carry-over of mycelia fragments during the preparation of spore fragments. No other data was excluded.
Replication	Each experiment was performed in at least two biological replicates. All replication attempts were successful. For each replicate, experimental and control groups were imaged or measured, processed and analyzed together.
Randomization	Respective strains for different experiments were selected randomly for inoculation from plate. All strains were grown under similar conditions and were therefore equivalent at the start of the experiment. The observed differences are due to the genotype of the analyzed strains.
Blinding	This was not necessary for this study. Strains were grown under similar conditions and were therefore equivalent at the start of the experiment. The observed differences can be attributed to the genotype.

Reporting for specific materials, systems and methods

We require information from authors about some types of materials, experimental systems and methods used in many studies. Here, indicate whether each material, system or method listed is relevant to your study. If you are not sure if a list item applies to your research, read the appropriate section before selecting a response.

Materials & experimental systems

n/a	Involved in the study	
☐	☒	Antibodies
☒	☐	Eukaryotic cell lines
☒	☐	Palaeontology and archaeology
☒	☐	Animals and other organisms
☒	☐	Human research participants
☒	☐	Clinical data
☒	☐	Dual use research of concern

Methods

n/a	Involved in the study	
☒	☐	ChIP-seq
☒	☐	Flow cytometry
☒	☐	MRI-based neuroimaging

Antibodies

Antibodies used	anti-FtsZ antibody produced in rabbit (1:200 dilution), Cambridge Bioscience<br>anti-WhiA antibody produced in rabbit (1:100), Cambridge Bioscience<br>anti-SsgB antibody, produced in rabbit (1:100 dilution), Cambridge Bioscience<br>anti-mCherry antibody, (1:200), Abcam 183628<br>anti-GFP, (1:500), Sigma SAB4301138<br>anti-rabbit secondary antibody detection module, ProteinSimple DM-001<br>Note: anti-FLAG antibody was conjugated to magnetic beads as part of the µMACS epitope tag protein isolation kit (FLAG 130-101-591). No Western blots with anti-FLAG antibody were performed.
Validation	Validation of commercially available antibodies was based on technical data sheets provided by the manufacturer:<br>anti-mCherry: https://www.abcam.com/mcherry-antibody-ab183628.html<br>anti-GFP: https://www.sigmaaldrich.com/GB/en/product/sigma/sab4301138?gclid=CjwKCAjwzt6LBhBeEiwAbPGOgc9RBS9UApzPwJfZpTt-cUZJirCqALsWHtNdZOtvuniNr54x_TmHNxoCoyUQAvD_BwE<br>anti-rabbit: https://www.bio-techne.com/p/simple-western/anti-rabbit-detection-module_dm-001#product-datasheets<br>Custom-made antibodies anti-WhiA and anti-FtsZ were validated before in Bush et al, mBio 4, e00684-13 (2013).and Ramos-Leon et al. eLife 10, e63387 (2021), respectively. Anti-SsgB antibody was validated by automated Western blotting, testing crude cell lysates of the wildtype and ssgB-deficient cells, and purified recombinant SsgB in serial dilutions. The SsgB antibody specifically recognised bands of the correct size in wildtype cell extracts and on purified protein.
